# Histologic Disease Persists beyond Mucosal Healing and Could Predict Reactivation in Ulcerative Colitis

**DOI:** 10.3390/jpm14050505

**Published:** 2024-05-10

**Authors:** Lucrezia Laterza, Anna Chiara Piscaglia, Stefano Bibbò, Vincenzo Arena, Massimo Brisigotti, Giovanna Fabbretti, Maria Loredana Stefanelli, Valentina Cesario, Rossella Maresca, Andrea Poscia, Daniela Pugliese, Eleonora Gaetani, Alfredo Papa, Giovanni Cammarota, Alessandro Armuzzi, Antonio Gasbarrini, Franco Scaldaferri

**Affiliations:** 1 Centro per le Malattie dell’Apparato Digerente (CEMAD), Digestive Disease Center, Fondazione Policlinico Universitario “A. Gemelli” IRCCS, Università Cattolica del Sacro Cuore, 00168 Rome, Italyfranco.scaldaferri@policlinicogemelli.it (F.S.); 2 Endoscopy and Gastroenterology Unit, State Hospital, 47893 Cailungo, San Marino; 3 UOC di Gastroenterologia, Dipartimento di Scienze Mediche e Chirurgiche, Fondazione Policlinico Universitario “A. Gemelli” IRCCS, 00168 Rome, Italy; 4 Istituto di Anatomia Patologica, Dipartimento Scienze della Salute della Donna, del Bambino e di Sanità Pubblica-Area Anatomia Patologica, Fondazione Policlinico Universitario “A.Gemelli” IRCCS, 00168 Rome, Italy; 5 Department of Pathology, “Infermi” Hospital, 47923 Rimini, Italy; 6 Internal Medicine, State Hospital, 47893 Cailungo, San Marino; 7 Dipartimento di Medicina e Chirurgia Traslazionale, Università Cattolica del Sacro Cuore, 00168 Rome, Italy; 8 UOC ISP Prevention and Surveillance of Infectious and Chronic Diseases, Department of Prevention, Local Health Authority (ASUR-AV2), 60035 Jesi, Italy; 9 IBD Center, IRCCS Humanitas Research Hospital, 20089 Milan, Italy

**Keywords:** mucosal healing, histological healing, ulcerative colitis, relapse, prognosis

## Abstract

Mucosal healing (MH) is the main target in ulcerative colitis (UC) treatment. Even if MH lowers the risk of disease reactivation, some patients still relapse. Histologic activity (HA) beyond MH could explain these cases. This study aims to assess how many patients with MH have HA and which lesions are associated with relapse. We retrospectively enrolled UC patients showing MH, expressed as a Mayo Endoscopic Subscore (MES) of 0 and 1 upon colonoscopy. We reviewed the histological reports of biopsies evaluating the presence of typical lesions of UC and assessed the number of clinical relapses after 12 months. Among 100 enrolled patients, 2 showed no histological lesions. According to univariate analysis, patients with a higher number of histological lesions at the baseline had a higher risk of relapse (OR 1.25, *p* = 0.012), as well as patients with basal plasmacytosis (OR 4.33, *p* = 0.005), lamina propria eosinophils (OR 2.99, *p* = 0.047), and surface irregularity (OR 4.70, *p* = 0.010). However, in the multivariate analysis, only basal plasmacytosis (OR 2.98, *p* = 0.050) and surface irregularity (OR 4.50, *p* = 0.024) were confirmed as risk factors for disease reactivation. HA persists in a significant percentage of patients with MH. Despite the presence of MH, patients with basal plasmacytosis and surface irregularity have a higher risk of relapse.

## 1. Introduction

Ulcerative colitis (UC) is a chronic inflammatory disease involving the colonic mucosa, characterized by alternating periods of relapse and remission [1].

In the past, the main therapeutic goal was the management of symptoms such as stool frequency and rectal bleeding [2]. However, due to the typical superficial inflammation, mucosal healing (MH) has become a major therapeutic target in the era of biological therapy [3,4]. 

Endoscopic evaluation allows the assessment of multiple signs of mucosal inflammation and several endoscopic scores have been developed to standardize endoscopic language. The Mayo endoscopic subscore (MES) is one of the most common scores and categorizes mucosal inflammation from grade 0 to 3 based on the vascular pattern, erythema, friability, erosions, and ulcers of the colonic mucosa [5]. Grades 0 and 1 are generally accepted as the definition of MH, even if some authors have suggested a stricter definition of MH limited at grade 0 [6].

However, the definition of MH is still controversial. Literally, MH indicates the complete restitution of colonic mucosa to its normal condition but in clinical trials and clinical practice, we often accept broader definitions of MH, including all patients without erosions and ulcers, but showing minimal endoscopic signs of inflammation, such as the persistence of edema, reduced vascular pattern, and mucosal erythema [7]. Nevertheless, recent data demonstrated that the stricter the definition of MH, the better the mid-term clinical outcome [6,8]. As a matter of fact, Boal Carvalho P. et al., in a retrospective study, reported that patients with a MES of 0 could still relapse after one year of follow-up, even if in a smaller percentage than patients with a MES of 1 (11.5% vs. 27.3%, respectively, *p* = 0.022), suggesting that variables other than MH could determine the likelihood of relapse, such as the persistence of microscopic disease activity, beyond a normal macroscopic mucosa [6]. Comparable findings were attained in a study conducted in Spain, wherein the percentage of patients with a MES of 0 experiencing relapse at 6 months was significantly lower than MES 1 patients (9.4 vs. 36.6%, *p* < 0.001) [8]. Moreover, data reported that evidence of histological inflammation could persist in 16–100% of patients with MH [9,10,11] and could predict reactivation in these patients. 

Histological activity in inflammatory bowel disease is defined by a wide spectrum of typical histological lesions. Historically, the microscopic diagnosis of UC is based on the presence of ubiquitous crypt architectural distortion and transmucosal inflammatory infiltrate defined as presenting with basal plasmacytosis, eventually associated with cryptitis and crypt abscesses and mucin depletion (even if it is less specific). Among these typical histological findings, basal plasmacytosis offers the strongest predictive value in identifying ulcerative colitis [12]. Concurrently, mucin depletion was found to be correlated with clinical relapse in UC patients with a MES of 0 [13].

However, UC typical histological lesions frequently overlap with other diseases, such as infective ones [14]. The European Crohn’s and Colitis Organisation [ECCO] convened an expert panel to define them [15]. Mucin depletion represents a reduction in the number of goblet cells leading to a consequent reduction in mucin droplets in the crypt of the surface epithelium; basal plasmacytosis is defined as an increase in plasma cell number at the mucosa base; crypt distortion is characterized by the loss of parallel crypt architecture and increased branching or variation in crypt size and shape; acute inflammatory infiltrate is defined as a rise in neutrophils within the lamina propria, whereas chronic inflammatory infiltrate is defined as having an increase in lymphocytes in the same area; Paneth cell metaplasia is the anomalous localization of these cells in segments of the colon distal to the hepatic flexure; cryptitis indicates the presence of at least one neutrophil within the crypt epithelium, whereas a crypt abscess is defined as having neutrophils present in the crypt lumen; basal lymphoid aggregates refer to the nodular collection of lymphocytes in the mucosa, and finally, the term “surface irregularity” describes the wavy aspect of the colonic mucosa’s surface due to wide crypt mouths resembling villous projections, also known as a “villiform surface” [15]. A widely recognized definition for an increase in eosinophils is still lacking. Eosinophilic cryptitis means the presence of at least one eosinophil in the crypt epithelium; however, an eosinophilic crypt abscess is characterized by the presence of these cells within a crypt lumen in the absence of luminal neutrophils [15]. As of the present, within the context of UC, a standardized definition of histological healing remains controversial [16]. Nonetheless, data have reported that the minimum requirement for histological healing seems to be the resolution of the crypt architectural distortion and of inflammatory infiltrate [12]. 

Furthermore, several studies over the years have shown a high correlation between histological healing, reduction of length of hospitalization, the possibility of relapse, and a lesser use of corticosteroid [16,17]. However, the question of whether specific histological lesions can predict reactivation in patients with MH is unclear. 

The primary aim of this study was to evaluate the rate of clinical relapse at 12 months in patients with MH based on the presence of specific histological lesions. The secondary aims were to evaluate whether the presence of specific histological lesions could influence the relapse, irrespective of the baseline therapies, and to evaluate whether patients with a MES of 0 and MES of 1 showed different relapse rates.

## 2. Materials and Methods

### 2.1. Study Design and Patients

We retrospectively reviewed endoscopic databases from two centers (one primary care center, the State Hospital of the Republic of San Marino, Borgo Maggiore, Republic of San Marino, and one tertiary care center, the Agostino Gemelli Hospital, Rome, Italy), searching for all patients who had undergone endoscopic evaluation for a previous diagnosis of UC. We included patients with completed colonoscopies and multiple biopsies in at least three colonic districts: rectum, and left and right colon. In the case of a known history of left-side colitis or proctitis, we also included sigmoidoscopies with biopsies in the rectum and sigmoid colon. A follow-up period after colonoscopy of at least 12 months was required. Exclusion criteria were a history of previous colonic resection, diagnosis of unclassified inflammatory bowel disease or colonic Crohn’s disease, a new diagnosis of UC, colonic diverticular disease, primary sclerosing cholangitis associated IBD, and concomitant therapy using experimental drugs. We selected endoscopic reports showing mucosal healing, defined as a MES of 0 or MES of 1 [5]. Disease extension was evaluated based on a previous complete colonoscopy showing endoscopic activity. In the case of more than one available previous colonoscopy, the one showing the major disease extension was chosen. 

### 2.2. Clinical Assessment

The primary outcome was the rate of clinical relapse during the 12-month follow-up. We reviewed patients’ charts to collect patients’ medical history, extension of disease following the Montréal classification [18], concomitant medications, and clinical activity at the time of their colonoscopy (baseline) and after 12 months (time 1). We defined clinical remission as having a UC Clinical Activity Index (CAI) score ≤ 4 [19]. The UC CAI score defines clinical activity based on the number of stools weekly, blood in stools, the investigator’s global assessment of the symptomatic state, abdominal pain, temperature due to colitis, extraintestinal manifestations, erythrocyte sedimentation rate (ESR), and hemoglobin [19]. We defined clinical relapse as one or more of the following criteria being met at any moment during the 12-month follow-up: UC CAI > 4 and/or need for significant therapy changes (i.e., need for steroids, immunomodulators, or anti-TNFα) and/or hospitalization for UC.

### 2.3. Histologic Evaluation

For each selected exam, we reviewed the database to find the corresponding histological report and manually searched for reported histological lesions typically associated with UC: chronic inflammatory infiltrate, acute inflammatory infiltrate, basal lymphoid aggregates, basal plasmacytosis, lamina propria eosinophils, mucus depletion, crypt distortion, crypt depletion/atrophy, cryptitis/pericryptitis, crypt abscesses, erosions, Paneth cell metaplasia, stromal alterations, crypt branching, and superficial irregularity [12,20]. All biopsies were fixed in formalin, and sections were stained with hematoxylin and eosin. Pathologists with expertise in the digestive tract performed all the microscopic examinations. Histologic lesions were evaluated for each considered colonic district: rectum, and left and right colon. Then, patients showing a specific lesion in at least one district were considered positive for that specific lesion.

### 2.4. Statistical Analysis

The primary endpoint is considered merely descriptive. Thus, we estimated that enrolling 100 patients would obtain a percentage of clinical relapse of 20% with a confidence interval of 95% and a margin of error of 7.84%. The clinical and demographic characteristics of the enrolled sample were shown through descriptive statistics. Mainly, quantitative variables were represented through minimum, maximum, range, and mean and standard deviation. In addition, qualitative variables were described in terms of absolute and percentage frequency. Regarding the continuous variables, normality was verified via the Kolmogorov–Smirnov test. The primary endpoint was evaluated based on the percentage frequency of clinical relapse for any histologic lesion. Both the rate of clinical relapse and the predictive or protective value of multiple factors concerning the primary outcome were assessed using the Stata 14 Mac version. We calculated the odds ratio (OR) using the corresponding 95% confidence interval (CI) and *p*-value. All variables considered were first included in a univariate analysis to assess their association with clinical relapse. A univariate logistic regression model and t-test were used for dichotomous and continuous variables, respectively. The correlation between clinical relapse and the number of histologic lesions was evaluated using Pearson’s test. The factors that resulted as being significant (*p* < 0.25) for clinical relapse according to the univariate analysis were then analyzed via multivariate analysis. For the multivariate analysis, model building was based on a stepwise backward procedure, including only variables with *p* < 0.05 in the final model. *p*-values < 0.05 were considered significant for the analysis.

## 3. Results

We enrolled 100 patients who fulfilled all the inclusion criteria with no exclusion criteria met. All patients were in clinical remission at the time of their baseline endoscopy. The age range was between 18 and 79 years (mean 42.2 years). Patients were heterogeneous for baseline therapies. Patients’ characteristics at the baseline are shown in Table 1. 

**Figure 1:**
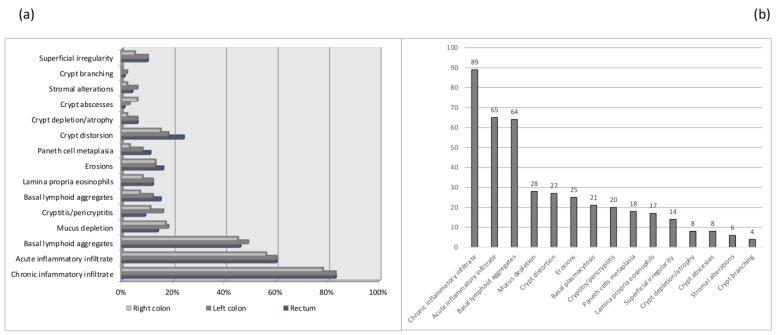
Distribution of histologic lesions at baseline for each considered colonic district (**a**) and globally (**b**).

Most of the patients were undergoing maintenance therapy with mesalazine (56%), whereas only 8% were being treated with methotrexate or were not receiving any therapy (defined as “others” in Table 1). At the baseline, only two patients with MH showed the complete absence of histologic lesions (“histological healing”). In all other cases, at least one histologic lesion was present, the most prevalent being chronic inflammatory infiltrates (89% of cases), acute inflammatory infiltrates (65% of cases), and basal lymphoid aggregates (64% of cases) (Figure 1a,b).

**Figure 2:**
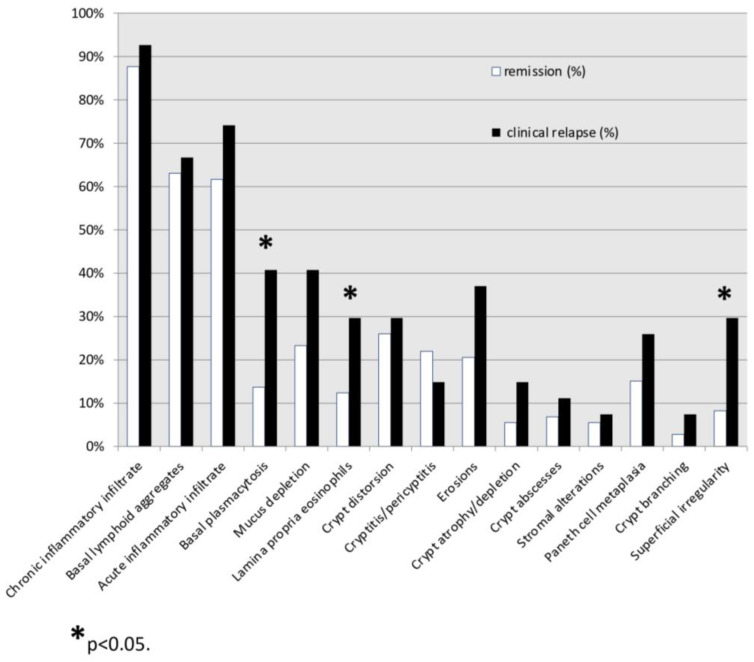
Distribution of histologic lesions at baseline in patients who will maintain remission and in patients who will relapse.

During the 12-month follow-up, 27% of the patients experienced a clinical relapse. The mean time for relapse was 6.5 months after their baseline endoscopy. Among the patients who experienced clinical relapse, the prevalence of histologic lesions at the baseline in at least one colonic district was as follows: chronic inflammatory infiltrate (93%), acute inflammatory infiltrate (74%), basal lymphoid aggregates (67%), mucous depletion (41%), basal plasmacytosis (41%), erosions (37%), crypt distortion (30%), lamina propria eosinophils (30%), surface irregularity (30%), Paneth cell metaplasia (26%), cryptitis/pericryptitis (15%), crypt depletion/atrophy (15%), crypt abscesses (11%), stromal alterations (7%), crypt branching (7%) (Figure 2). Instead, in patients who maintained remission, the prevalence was as follows: chronic inflammatory infiltrate (88%), basal lymphoid aggregates (63%), acute inflammatory infiltrate (62%), crypt distortion (26%), mucous depletion (23%), cryptitis/pericryptitis (22%), erosions (21%), Paneth cell metaplasia (15%), basal plasmacytosis (14%), lamina propria eosinophils (12%), surface irregularity (8%), crypt abscesses (7%), crypt atrophy/depletion (5%), crypt branching (3%) (Figure 2).

According to the univariate analysis, the age at the time of colonoscopy was a protective factor for clinical relapse; in fact, older patients showed a lower risk of relapse (OR 0.96, *p* = 0.028 [95% IC 0.93–0.99]). Similarly, the disease duration at the baseline was protective for clinical relapse (OR 0.90, *p* = 0.014 [95% IC 0.83–0.98]). Patients with a MES of 1 tended to have a higher probability of experiencing clinical relapse compared to patients with a MES of 0 (OR = 1.90, *p* = 0.188 [95% IC 0.72–5.15]). However, this difference was not statistically significant. Among the different histologic lesions, the presence of basal plasmacytosis at the baseline was a risk factor for clinical relapse (OR 4.33, *p* = 0.005 [95% IC 1.57–11.98]), such as lamina propria eosinophils (OR 2.99, *p* = 0.047 [95% IC 1.02–8.83]) and surface irregularity (OR 4.70, *p* = 0.010 [95% IC 1.45–15.22]). Patients with a higher number of histologic lesions at the baseline showed a higher probability of clinical relapse (OR 1.25, *p* = 0.012 [95% IC 1.05–1.49]). The mean number of histologic lesions in patients in remission was 3.7, whereas it was 5.2 in patients with clinical relapse (*p* < 0.05).

According to the multivariate analysis, the role of basal plasmacytosis and superficial irregularity was confirmed as risk factors for clinical relapse: OR 2.98, *p* = 0.050 [95% IC 1.00–8.87] and OR 4.50, *p* = 0.024 [95% IC 1.22–16.61], respectively. Instead, the presence of lamina propria eosinophils did not significantly increase the risk of relapse. The disease duration at the time of colonoscopy confirmed its protective role for future clinical relapse, with patients with a longer disease being at lower risk (OR 0.90, *p* = 0.024 [95% IC 0.82–0.98]).

## 4. Discussion

In UC, patients with MH showed better outcomes in terms of relapse and risk of cancer [21,22]. However, in long-term follow-up, some patients relapsed despite the achievement of endoscopic MH. In fact, beyond MH, patients with histologic healing showed better outcomes, with a lesser use of corticosteroid, a lower colon rectal cancer risk, and fewer colectomies [16]. Based on this data, histologic healing has been recently proposed as a different and more ambitious therapeutic target in UC [23,24]. However, despite its advantages, it has not yet been used as a routine clinical practice therapeutic target [4]. Furthermore, a universally accepted definition of histologic healing is lacking [25].

In this retrospective study, we hypothesized that HA beyond MH could be responsible for relapse in many patients. We evaluated the prevalence of the persistence of microscopic activity in patients with macroscopically normal mucosa (MES of 0) or with minimal endoscopic activity (MES of 1), which are both included in the definition of MH used in clinical practice. In our cohort, only two patients showed the complete absence of histological activity, in line with the literature, reporting the persistence of HA in 16–100% of patients [11,26], confirming that endoscopic remission does not always correspond to histologic remission in UC [27,28]. In a retrospective study conducted by Christensen et al., MH was not able to predict relapse-free survival, whereas the persistence of HA beyond MH was associated with a higher risk of relapse in a large cohort of 646 patients. Particularly, histologic normalization (completely normal mucosa with no features of chronicity present) was associated with increased odds of relapse-free survival compared both with quiescence (crypt atrophy or branching without signs of active inflammation including erosions, abscesses, or focal neutrophil infiltration) and with active disease (epithelial infiltration by neutrophils, crypt abscesses, erosions, or ulceration) [29]. However, this study did not investigate the role of specific histologic lesions in predicting relapse. Similar results are reported in another prospective cohort study. Defining histologic remission in this paper as having a score on the Nancy histologic index (NHI) < 2, the authors found a significant prolonged relapse-free survival rate in those patients with a MES of 0 who achieved histologic remission [30], suggesting a superior role of histological assessment than solely using endoscopy as a therapeutic target. In addition, in a recent systematic review and meta-analysis, Yoon et al. recently found that among patients with endoscopic remission, defined as a MES of 0, those who achieved histological remission had a 63% lower risk of clinical relapse compared to patients with persistent histologic activity (relative risk, 0.37; 95% CI, 0.24–0.56) [31]. 

In our cohort, we decided to include patients undergoing multiple biopsies in more than one colonic district because microscopic inflammation could be patchy, especially after therapy [24,32]; thus, biopsies limited to the rectum could miss significant inflammatory signs. Identifying histologic lesions that predict clinical relapse could be helpful for clinicians to recognize patients deserving shorter follow-up or therapy escalation, notwithstanding a clinical and endoscopic remission. In our cohort, according to the univariate analysis, basal plasmacytosis, lamina propria eosinophils, and superficial irregularity were associated with a higher relapse rate during the 12-month follow-up. However, in the multivariate analysis, only basal plasmacytosis and superficial irregularity confirmed this negative prognostic value.

Basal plasmacytosis, meaning the presence of a dense infiltrate of plasma cells in the mucosal lower third [20], has already been demonstrated in other cohorts to predict clinical relapse. In fact, Bessissow et al. demonstrated that basal plasmacytosis was predictive of clinical relapse in a cohort of 75 patients, of which 40% were being treated with infliximab or vedolizumab [33]. Similarly, Bitton et al. demonstrated that the presence of basal plasmacytosis in rectal biopsies was associated with a shorter time to relapse [34]. Our data confirm the role of basal plasmacytosis (OR 2.98, *p* = 0.050 [95% IC 1.00–8.87]) in identifying patients with a major risk of relapse, even in patients with different characteristics. In fact, including patients not only from a referral center but also from a primary care center, our population is more representative of the complex characteristics of UC patients in real life, also including patients undergoing different therapies that have no influence on the clinical relapse rate in the presence of basal plasmacytosis. Furthermore, only patients with endoscopically inactive disease were included in the previously mentioned studies. Instead, we decided to also include patients with mildly active endoscopic disease (MES of 1) because in the current practice and clinical trials, mild disease is generally included in a broad definition of “MH”, and it is not considered a reason to change therapy. In our cohort, the presence of basal plasmacytosis or superficial irregularity could predict relapse irrespective of the macroscopic mucosal appearance; in fact, there were no significant differences in the clinical relapse rate between patients with a Mayo endoscopic subscore of 0 and 1, underlying the importance of histologic control of the disease, beyond the endoscopic aspect. 

However, the physiopathogenesis of basal plasmacytosis in predicting clinical relapse is still unclear. In fact, basal plasmacytosis is generally considered a sign of chronicity and not a sign of activity, differently from lamina propria eosinophils and neutrophils, and is considered to be a key feature in IBD diagnosis [12,20]. 

Interestingly, we also identified superficial irregularity as being predictive of relapse (OR 4.50, *p* = 0.024, [95% IC 1.22–16.61]). Superficial irregularity is defined as an “undulating or broadly villiform surface” by the British Society of Gastroenterology [20]. It is an alteration related to the separation of crypts, with consequently wide crypt mouths conferring a profile like a villous projection to the mucosa. Even if the role of this alteration in predicting relapse is still not clear, it could probably configure a deep alteration in the colonic mucosal architecture, implying a severe gut barrier dysfunction that could be related to a more aggressive disease [35].

Regarding other histologic lesions, an eosinophilic infiltrate in the lamina propria was more frequently observed at the baseline in patients relapsing after 12 months (30%). In addition, it was a predictive factor for relapse in the univariate analysis. However, the multivariate analysis did not confirm this role. Further larger studies are required to investigate the role of this lesion adequately. In fact, previous small studies suggested that it could be a negative prognostic factor [36,37]. In our cohort, we did not find any differences between relapsing and non-relapsing patients regarding the other observed histologic lesions, even if some of them were previously reported as being associated with clinical relapse. Particularly, an acute inflammatory infiltrate, that has been suggested as an important prognostic factor [9,13] was not associated with a higher relapse rate in our cohort.

Interestingly, in our cohort, there were no significant differences between a MES of 0 and 1 in terms of the probability of relapse, notwithstanding a trend of higher relapse in patients with a MES of 1. These data suggest that HA could be more important and significant in UC patients as a prognostic factor, compared to endoscopic appearance. In fact, in patients who relapsed, we observed a different distribution of histologic lesions in relation to the MES. The incidence of basal plasmacytosis was 38.9% and 44.4% in patients with a MES of 0 and 1, respectively. Meanwhile, the surface irregularity showed a prevalence of 11.1% and 66.7% in MES 0 and MES 1 patients.

However, this study presents some limitations. We did not use a quantitative system of histological measuring. We decided to describe the single primary lesions that are generally considered to calculate the activity scores but also included generally excluded lesions to obtain a more comprehensive description of histological characteristics at the baseline. In fact, multiple histological scoring systems exist for UC, with no universally accepted method of assessing activity in biopsies [38], and all the scores showed some pros and cons. The Geboes score focuses on polymorphonuclear/neutrophil infiltrates and epithelial damage in different grades, generating a 29-order scale that, even if largely used in clinical practice, could be too complex and difficult to be applied by non-expert pathologists [39]. The Robarts Histopathology Index is a 33-grade score based on an evaluation ranging from 0 to 3 of four typical histological lesions: inflammatory infiltrates, neutrophils in the lamina propria, neutrophils in the epithelium, and erosions/ulcerations. Each lesion showed a different weight, expressed with the use of different multiplicative factors: 1, 2, 3, and 5, respectively [40]. The Nancy Index provided a more simplified evaluation of histologic activity, including only three typical lesions (chronic and acute inflammatory infiltrates and ulceration) with a range of 0 to 4 of grading for each one [41]. Even if all of these three main histological scores prove to be accurate in identifying disease activity modifications, ECCO only recommends the Robarts Histopathology Index (RHI) and the Nancy Histological Index (NHI) for the evaluation of histologic disease activity [24,42]. However, the aim of these indices is to have a common measure of HA in multicenter clinical trials and a reliable tool to measure the different efficacy of different drugs in reducing HA over time, but they do not focus on the overall severity of organ damage or on the disease duration; thus, they only give a single-time snapshot of disease activity, without providing data about the risk of relapse of that single patient. Thus, the choice of one score could limit our view of the real histologic scenario of enrolled patients. For this reason, in our cohort, we decided to include also lesions that are not included in these scoring systems, and we demonstrated that basal plasmacytosis and surface irregularity could be more helpful in predicting relapse compared to lesions evaluated in scoring systems (i.e., chronic and acute inflammatory infiltrates), as they probably reflect a more aggressive underlying chronic activity and deeper damage in the gut barrier. 

Furthermore, in clinical practice, histologic scores are not extensively used, and the common practice for clinicians is to face the interpretation of qualitative histologic reports based on the description of single histologic lesions. In this context, our data could provide a useful tool in clinical practice as we provide evidence supporting a prognostic evaluation based on qualitative descriptions of histological lesions.

Another limit of our work is that in our cohort, the analysis of the histologic report was retrospective, and more than one pathologist performed the microscopic evaluation. Even if all the pathologists were experts in gastrointestinal pathology, the interobserver variability in the description could not be excluded. In fact, no clear thresholds for normal lamina propria inflammatory cells are reported in the literature [38], and basal plasmacytosis and lamina propria eosinophils have been reported to show poor interobserver agreement [15,42], leading to a poor applicability of our data in a clinical setting. However, in the future, this scenario could deeply change due to the new perspectives related to the application of artificial intelligence, even in histological evaluation. In fact, a new histological score, the PICaSSO Histologic Remission Index (PHRI), based only on neutrophils, has been recently described that could be implemented in a computer-aided diagnosis system that could be trained using a deep learning strategy to detect neutrophils, to calculate the PHRI, and to distinguish active from quiescent UC. The score evaluates the presence of neutrophils in the lamina propria (graded from 0 to 1 if absent or present, respectively) and in the epithelium, which are furtherly classified as neutrophils in the surface epithelium (1 point), neutrophils in the cryptal epithelium configuring cryptitis (1 point), and neutrophils in crypt abscesses (1 point). In the case of the absence of an intact surface epithelium in the biopsies, as for example in the case of erosion/ulceration, 1 point is scored. The total score, deriving from the sum of the above, could be maximum of 4. This new score is significantly correlated with clinical outcomes such as hospitalization, colectomy, and major treatment changes in a 12-month follow-up [43].

Another limit of our study is that, in our cohort, we were not able to integrate data about C-reactive Protein (CRP), ESR, and fecal calprotectin with histological ones, as they were not available for all patients, and the times of collection were inconsistent among patients due to the retrospective nature of the study. Thus, no analysis was possible regarding the value of these markers in predicting disease relapse.

## 5. Conclusions

Our study suggests always searching for histologic activity in UC patients with endoscopic MH, as it is the real risk factor for clinical relapse in apparently inactive disease, by using multiple biopsies or, in the future, with the use of virtual chromoendoscopic, confocal endomicroscopy or with artificial intelligence, trained to identify the main histologic activity factors for relapse. This approach could help to identify patients with a higher risk of relapse, hence deserving more intensive monitoring of diseases or optimization of therapy to achieve histological healing. From a forward-looking perspective and within the realm of increasingly personalized medicine, it would be compelling to observe how the various classes of drugs impact histologic activity and how the attainment of complete histologic healing is associated with better long-term outcomes such as colon rectal cancer development. 

## Figures and Tables

**Table 1:** Patients’ baseline characteristics.

Baseline Patients Characteristics	Total (n = 100)	Patients in Remission (n = 73)	Patients with Clinical Relapse (n = 27)
Gender (%)			
M	49%	51%	44%
F	51%	49%	56%
Age (years, mean ± SD)	42.2 ± 14.6	44.1 ± 14.8 *	37.1 ± 12.9 *
Disease duration (year, mean ± SD)	9.1 ± 9.0	10.5 ± 9.9 *	5.3 ± 4.2 *
Therapies (%)			
5-ASA	56%	53%	63%
Steroids	6%	7%	7%
Anti-TNFα	15%	14%	19%
Thiopurines	13%	15%	7%
Combo (thiopurines + antiTNFα)	2%	1%	4%
Others	8%	8%	0%
Disease extension following Montréal classification (%)			
E1	20%	22%	15%
E2	40%	36%	52%
E3	40%	42%	33%
Mayo endoscopic subscore			
0	76%	79%	67%
1	24%	21%	33%

* Differences between patients with clinical relapse and remission are statistically significant (*p* < 0.05).

## Data Availability

All data generated or analyzed during this study are included in this article. Further inquiries can be directed to the corresponding author.
